# Ectonucleotidases in Blood Malignancies: A Tale of Surface Markers and Therapeutic Targets

**DOI:** 10.3389/fimmu.2019.02301

**Published:** 2019-10-04

**Authors:** Tiziana Vaisitti, Francesca Arruga, Giulia Guerra, Silvia Deaglio

**Affiliations:** Department of Medical Sciences, University of Turin, Turin, Italy

**Keywords:** CD38, CD39, CD73, leukemias, myeloma, lymphoma, immunosuppression, tumor microenvironment

## Abstract

Leukemia develops as the result of intrinsic features of the transformed cell, such as gene mutations and derived oncogenic signaling, and extrinsic factors, such as a tumor-friendly, immunosuppressed microenvironment, predominantly in the lymph nodes and the bone marrow. There, high extracellular levels of nucleotides, mainly NAD^+^ and ATP, are catabolized by different ectonucleotidases, which can be divided in two families according to substrate specificity: on one side those that metabolize NAD^+^, including CD38, CD157, and CD203a; on the other, those that convert ATP, namely CD39 (and other ENTPDases) and CD73. They generate products that modulate intracellular calcium levels and that activate purinergic receptors. They can also converge on adenosine generation with profound effects, both on leukemic cells, enhancing chemoresistance and homing, and on non-malignant immune cells, polarizing them toward tolerance. This review will first provide an overview of ectonucleotidases expression within the immune system, in physiological and pathological conditions. We will then focus on different hematological malignancies, discussing their role as disease markers and possibly pathogenic agents. Lastly, we will describe current efforts aimed at therapeutic targeting of this family of enzymes.

## Introduction

Adenosine triphosphate (ATP) and nicotinamide dinucleotide (NAD^+^) are present at millimolar concentrations inside the cell, where they represent the currency for energy metabolism. In the extracellular space their concentrations are usually low, but can transiently increase through active or passive mechanisms. Specifically, active release can occur via exocytosis or can be mediated by selective transmembrane proteins (e.g., pannexin, connexin). Otherwise, extracellular ATP can derive from passive leakage from necrotic or injured cells. Both mechanisms are enhanced during inflammation, hypoxia and cancer (1–3). High levels of extracellular ATP and NAD^+^ behave as danger signals, alerting the immune system to possible tissue damage. To prevent protracted reactions leading to chronic inflammation, homeostasis is rapidly restored through a scavenging circuit operated by nucleotide-catabolizing enzymes that produce the immunosuppressant adenosine and inosine, which can re-enter the cell, reconstituting the nucleotide pool (4).

**Figure 1:**
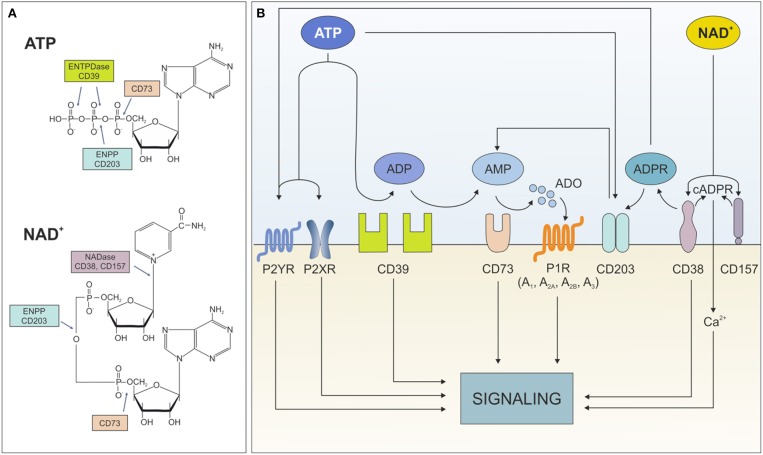
Ectonucleotidases network. **(A)** Structure of ATP and NAD^+^ with indication of cleavage sites by the different ectonucleotidases. **(B)** Schematic representation of the fate of extracellular ATP and NAD^+^ dismantling operated by the different ectonucleotidases and the functional consequences of the generated metabolites. ENTPDase, ecto-nucleoside triphosphate diphosphohydrolase; ENPP, ecto-phosphodiesterase/nucleotide phosphohydrolase; ATP, adenine-triphosphate; ADP, adenine-diphosphate; AMP, adenine-monophosphate; ADO, adenosine; P2XR, ATP-gated P2X receptor cation channel; P2YR, G protein-coupled P2Y receptor; P1R, adenosine purinergic receptor; NAD, nicotinamide adenine dinucleotide; ADPR, ADP-ribose; cADPR, cyclic-ADPR; Ca^2+^, calcium ions.

Extracellular ATP influences cell metabolism, adhesion, and migration in settings of acute inflammation (5). Likewise, extracellular NAD^+^ is critical in immune system homeostasis by regulating homing processes of different subsets of immune cells (6). Both nucleotides exert these effects through binding surface purinergic receptors of the P2X and P2Y families. An extensive overview of their expression in different cell types and their role in healthy and pathological conditions can be found in Burnstock (7) and Di Virgilio et al. (8). Beside receptor-mediated signaling, which will be not addressed in the current review, extracellular ATP and NAD^+^ can be degraded by an integrated network of ectonucleotidases, which generate intermediates that modulate signaling and activate immunoregulatory circuits. To date, eight different ectonucleoside triphosphate diphosphohydrolase (ENTPDase) subtypes have been identified (ENTPD1-8, each with distinct catalytic properties and tissue-specific distribution) (9, 10). The best characterized ATP degrading pathway proceeds through the sequential action of CD39 (ectonucleoside triphosphate diphosphohydrolase-1, ENTPD-1), which converts extracellular ATP (or ADP) to adenosine monophosphate (AMP), and CD73 (5'-nucleotidase) that converts AMP to adenosine (ADO). As an alternative, ADO can be generated from NAD^+^ through to the coordinated action of CD38 (NAD glycohydrolase), which generates ADP ribose (ADPR), and PC-1 (ectonucleotide pyrophosphatase/phosphodiesterase family member 1), which generates AMP. The removal of the final phosphate occurs via CD73, which consequently acts as bottleneck enzyme for both cascades (11). It is noteworthy to mention that lack of other key nucleotide-metabolizing enzymes, including NTPDase or CD38, can globally impact on ATP/NAD dismantling. Furthermore, not all extracellular NAD^+^ is converted to ADO: ADP ribose (ADPR) and—to a lesser extent—cyclic ADPR (cAPDR), generated by CD38, elicit signal transduction by modulating intracellular calcium levels (Figure 1).

Despite being a physiological way to prevent excessive inflammation and tissue damage, in tumor biology ectonucleotidases can contribute to generate immunosuppressive conditions (4).

Similar to other mechanisms that are hijacked to serve pro-tumor purposes, tumor cells take advantage of the expression of these molecules. Levels of extracellular nucleotides rise in response to hypoxia and ischemia, two defining feature of the tumor environment (12, 13). In cancer, including hematological malignancies, over-expression of ectonucleotidases has been linked to increased homing to protected niches, increased survival and proliferation, as well as modulation of immune responses toward tolerance. In some instances, ectonucleotidases have become reliable markers to monitor disease and to stratify patient subsets or molecular targets for novel treatment strategies (14).

The aim of this review is to provide an overview of the structure and function of these enzymes in physiological conditions and then to focus on their expression and role in several hematological diseases. The reader should take in consideration that while CD38 has been extensively studied from the biological standpoint over the years and is now widely adopted as a marker in the clinical practice, the enzymes of the ATP-dismantling cascade gained visibility in the field of hematological malignancies only recently. For this reason, most of the notions concerning CD39 and CD73 reported in the present review are based on experimental rather than clinical experience.

## CD38 and CD157

### Structure and Expression

Human CD38 and CD157/BST-1 are part of the same family of NADase/ADPR cyclase enzymes and share several features that reflect their origin from a common ancestor, a soluble enzyme found in the sea mollusk *Aplysia californica* (15, 16). Consistently, analysis of the coding genes shows a high degree of similarity in terms of exon-intron structure to the *Aplysia* ADP Ribosyl Cyclase (*ADPRC*) gene. Moreover, phylogenic analysis highlighted that *CD38* and *CD157* clearly derived from gene duplication, an event happened millions of years ago (17). During evolution from the original ancestral gene, CD38 and CD157 molecules acquired novel characteristics, including cell surface localization (18).

CD38 is a surface glycoprotein characterized by a relatively large extracellular domain that harbors the catalytic site, a single transmembrane pass and a short cytoplasmic tail (19). CD157 on the contrary, is attached to the membrane via a glycosylphosphatidylinositol (GPI) anchor (20). The extracellular domain of both molecules contains conserved critical residues that are essential for the enzymatic activity (21–24).

CD38 and CD157 pattern of expression is distinct in most tissues, including the hematopoietic system, suggesting that they regulate different cellular functions. Specifically, within the immune system, CD38 expression is high in immature hematopoietic cells, as well in activated T, B, dendritic and natural killer cells, but it is down-modulated in mature lymphocytes (20). CD157 on the other hand is mainly expressed by cells of the myelomonocytic lineage, including neutrophils, eosinophils, basophils, monocytes, macrophages, and plasmacytoid dendritic cells (20) (Table 1).

**Table 1:** Pattern of ectonucleotidases expression in non-malignant blood cells.

**Antigen**	**Cell population**	**Expression levels**	**References**
**CD38**			(18, 20)
	**B cells**		
	Progenitors	++	
	Naive	±	
	Naive activated	+	
	GC B cells	++	
	Post-GC	+	
	Memory B cells	-	
	Plasmablast	++	
	Plasmacell	+++	
	**T cells**		
	Naïve	+	
	Stem cell memory	±	
	Activated	+	
	**NK cells**	++	
	**Myeloid cells**		
	Inflammatory MF	+++	
			
	**Dendritic cells**		
	Immature	±	
	Mature	+	
**CD157**			(25)
	**B cells**		
	Progenitors	+	
			
	**T cells**		
	Pre-T cells	+	
	**Myeloid cells**		
	Precursors	+	
	Monocytes	+++	
	Macrophages	+++	
	Neutrophils	++	
	Eosinophils	++	
	Basophils	++	
**CD203a**			(26)
	**B cells**		
	Plasmacells	++	
**CD39**			(27)
	**B cells**		
	B regs	+++	
	**T cells**		
	CD4^+^	±	
	CD8^+^	±	
	Tregs	+++	
	Th17	++	
	**NK cells**	±	
	**Myeloid cells**		
	Monocytes	+++	
	Myeloid-derived suppressor cells		
	PMNs	+++	
	Macrophages M2	++	
**CD73**			Reviewed in (27)
	**B cells**	+++	
	**T cells**		
	CD4^+^	++	
	CD8^+^	++	
	Tregs	+++	
	Th17	++	
	**Myeloid cells**		
	Myeloid-derived suppressor cells	+	
	Macrophages M2	+	

*Expression of ectonucleotidases in B and T lymphocytes, myeloid components and natural killer cells*.

### Functions of CD38 and CD157

CD38, the main mammalian member of the ADPRC family, is a multifunctional ectoenzyme, involved in the catabolism and degradation of extracellular NAD^+^ (under normal pH) and NADP^+^ (under acidic pH). The main catalytic activity is that of NAD^+^ glycohydrolase that generates nicotinamide and ADPR. A minor activity of CD38 is that of NAD cyclase, which generates cADPR, then hydrolyzed to ADPR. Lastly, in the presence of NADP^+^ as substrate and of nicotinic acid and under acidic pH levels, CD38 can generate NAADP [revised in (28)]. ADPR, the main product of all these activities, undergoes different fates: (i) it can be covalently attached to target proteins modifying their functions, a process mediated by ADP-ribosyltransferases (ARTs) and known as ADP-ribosylation; (ii) it may bind purinergic receptors, specifically P2Y1, serving as a signaling molecule (29, 30); (iii) alternatively, it can enters the cells, where it can activate Ca^2+^ channels, activating calcium-induced calcium release. The calcium mobilizing property of ADPR is shared with the other products originated by CD38 activities, including cADPR and NAADP (31, 32). cADPR binds to ryanodine receptors (RyR) expressed on the endoplasmic reticulum, ADPR binds to membrane melastatin-related transient receptor potential cation channels TRPM2 (33) and NAADP binds to receptors expressed by acidic organelles, such as lysosomes, suggesting a role as calcium messenger in the endocytic pathway (32). Therefore, the final result of CD38 enzymatic activities is the modulation of intracellular calcium levels, which contribute to cell homeostasis and signaling (34). Similar to CD38, CD157 is able to generate cADPR and subsequently ADPR when incubated with NAD^+^, suggesting that this molecule has ADPRC and cADPR hydrolase activities (35, 36). However, its catalytic efficiency is markedly lower than that of CD38 (37).

The position of CD38 and CD157 on the cell surface suggests that their enzymatic activities can be modulated through protein-protein interactions. For example, CD38 can interact with CD31, a non-substrate ligand (38) and can form strong lateral association within lipid rafts. In T lymphocytes CD38 localizes to the immune synapse in close contact with the T cell receptor (39). Similar observations of a critical localization of CD38 in the “center of action” were reported for B lymphocytes, monocytes and NK cells (39–42). Although the functional implications of these interactions remain largely unknown, it is conceivable that CD38 may somehow act as a signaling booster, providing the additional increase in intracellular Ca^2+^ levels that may be necessary to induce or maintain receptor-mediated signaling.

CD38 has been implicated in T and B cell maturation, proliferation and cytokine production, mainly through the activation of intracellular signaling cascades that include phosphorylation of Zap70, c-Cbl, PLC-gamma, PI-3K, and MAPK (43). In addition, CD38 appears a critical mediator of chemotaxis and homing of immune cells. Genetic evidence was obtained from experiments performed using CD38^−/−^ mice, showing that dendritic cells and neutrophils of these animals have defective ability to migrate toward inflammation sites in response to specific chemokines, mainly CCL2, CCL19, CCL21, and N-Formylmethionine-leucyl-phenylalanine (fMLP). The underlying mechanism resides in the loss of cADPR production by CD38, thereby limiting calcium mobilization upon engagement of the chemokine receptors (41, 44, 45). Similar to CD38, also CD157 is involved in chemotaxis and trans-endothelial migration of neutrophils (46).

## CD203a/PC-1

Plasma cell antigen 1 (PC-1) is a class II transmembrane glycoprotein, belonging to the ecto-nucleotide pyrophosphatases/phosphodiesterases (E-NPPs) family that comprises seven different members. It is expressed on the cell surface and in different cellular compartments, including the endoplasmic reticulum. It is a homodimer characterized by a short amino-terminal intracellular portion, a single transmembrane sequence and a large extracellular domain, containing the catalytic site of the molecule. PC-1 has been detected in almost all tissues. In the immune system, its expression is restricted to plasma B cells, where it has been considered for several years as a marker (47).

E-NNPs, including PC-1, are part of the network of surface ectonucleotidases involved in several physiological functions, including nucleotide recycling, tuning of purinergic receptor signaling, modulation of pyrophosphate levels (critical for physiological calcification) and regulation of chemotaxis (10, 48). They exert their enzymatic functions by hydrolyzing the 5′-phosphodiester bonds in nucleotides and their derivatives, leading to the formation of nucleoside mono- or di-phosphate while releasing pyrophosphate (PPi) (Figure 1). Recent data prompted the idea that E-NNPs are part of the enzymatic cascade that produces ADO starting from ATP and NAD^+^, through the generation of AMP which is in turn the substrate for CD73, releasing ADO (10).

## CD39 and CD73

### Structure and Enzymatic Activity

The cascade starting from ATP and leading to ADO production is governed by CD39 and CD73 and affects purinergic signaling by modulating ligand availability.

CD39, also known as NTPDase1, belongs to the (ecto-)nucleotide triphosphate diphosphohydrolase [(E)NTPDases] family of ectoenzymes that includes eight members differing for their catalytic properties and for their cellular localization. It is encoded by the *ETNPD1* gene and it was the first NTPDase to be cloned and sequenced. Different splicing products have also been identified.

Together with NTPDase2, 3, and 8, CD39 has the active site facing the extracellular space. This site contains the “apyrase conserved regions,” highly conserved sequence domains, which are required for the phosphohydrolysis of extracellular nucleotides. Distinct phosphohydrolytic activities among ENTPDase family members are due to substantial differences in their sequences, which reflect in secondary, tertiary and quaternary structural differences (49). Consequently, they have distinct preferences for substrates and divalent cations, hydrolyze nucleoside triphosphates at varying rates, and generate different products. Micromolar levels of Ca^2+^ or Mg^2+^ ions are absolutely required for these four cell-surface-located ectoenzymes to exert maximal activity. CD39/ENTPD1, with a preference of Mg^2+^ over Ca^2+^, equally degrades ATP and ADP.

Other NTPDase are located inside the cells or toward the lumen of intracellular organelles. At variance with other NTPDases, CD39 can hydrolyze both ATP and ADP thus representing the rate-limiting enzyme in AMP production. A recent general description of CD39 is reviewed in Allard et al. (27).

Several structural requirements control the activity of this enzyme. First, two transmembrane domains are essential to anchor the protein to the cell membrane and to maintain the catalytic activity, as well as substrate specificity (50). Second, post-translational modifications, such as proteolysis and glycosylation, make the enzyme fully functional. Third, palmitoylation of the N-terminal intracytoplasmic domain enables association of CD39 with the lipid rafts, another requirement for full CD39 activity (51, 52).

Whereas, CD39 catalyzes the hydrolysis of ATP to AMP, CD73 is the rate-limiting enzyme in ADO generation pathways and it represents the step where NAD^+^ and ATP degradation cascades can converge. CD73 belongs to the ecto-5′-nucleotidase family that catalyzes the hydrolysis of 5′-AMP to ADO and inorganic phosphate (53, 54). It is encoded by the *NT5E* gene and is a GPI-anchored protein of ~70 kDa. This enzyme also exists in a soluble form derived from shedding of the GPI anchor and maintaining a similar enzymatic activity (55). The structure of CD73 is organized in three domains: a N-terminal domain with metal-binding sites, a C-terminal domain where the catalytic site is located, and a bridge alpha helix domain. Post-translational glycosylation, resulting in different molecular weight glycoforms, has also been reported (56). Full catalytic activity requires CD73 homodimerization, stabilized by non-covalent hydrophobic interactions between adjacent C-terminal domains, as well as the binding of two zinc ions. CD73 homodimers cycle from through open and closed conformation for efficient AMP hydrolysis (57). Other forms of 5'- nucleotidase exist in the cytoplasm and lysosomes and can be distinguished from CD73 by their substrate affinities, requirement for Mg^2+^ ion, activation by ATP, and inhibition by inorganic phosphate.

Furthermore, both CD39 and CD73 are characterized by specific structural features important for their potential activity as signal transducers, independent of their role as ATP-dismantling enzymes. For instance, CD39 shows sequence motifs, as well as secondary and tertiary structure similarities, with members of the actin/HSP70/sugar kinase superfamily (58) and can directly interact with Ran-binding protein M (RanBPM), a membrane scaffolding protein with GTPase activity (59). Likewise, CD73 can act as a cell adhesion molecule (59, 60) and can interact with and activate specific G-protein coupled receptor (GPCR) to increase intracellular cAMP levels, with reported effects on cancer cell aggressiveness, metastasis and angiogenesis [reviewed in (61, 62)].

### Expression and Functions in Non-neoplastic Conditions

In human peripheral blood, the majority of B cells and monocytes express CD39, as roughly one third of CD4^+^ T cells and, in a very small proportion, CD8^+^ T cells and NK cells. On the contrary, CD73 is expressed by B cells and by ~50% of CD8^+^ T cells, but only by ~10% of CD4^+^ T cells and <5% of NK cells [reviewed in (27)]. NK cells can acquire CD73 expression upon contact with mesenchymal stem cells and can then contribute to immune response modulation by converting AMP to ADO (63).

CD39 was initially described as a B cell activation marker (64), although, functionally, CD39 deletion did not impair global B cell number, but rather affected B cell memory responses to T-dependent antigens. This suggested that CD39 contributes to affinity maturation of antibody response and to post-germinal center B cell differentiation (65). Human B cells co-express CD39 and CD73, as well as adenosine receptors and they benefit from the generated ADO through an autocrine mechanism. The activation of adenosine receptors favors expansion and functions of adenosine-producing B cells. Furthermore, ADO generated by B cells influences the behavior of neighboring T cells (66). It was also observed that ectonucleotidases expression is associated with high proliferative capacity of regulatory B cells (Bregs) and with increased secretion of IL-10 and IL-6, which in turn potentiate the immunosuppressive activity toward T cells (67). Moreover, CD73 expression and adenosine production are involved in class-switch recombination. Indeed, CD73 lacking B cells presented impaired maturation of antibodies production (68).

Within the T cell compartment, CD39 is mainly expressed by CD4^+^ cells and principally by Tregs and Th17 even though it was reported on effector memory cells as well. Generally, expression of CD39 associates with the acquisition of an immunosuppressive phenotype and increased susceptibility to apoptosis and metabolic stress, therefore representing a mechanism to control expansion of cytotoxic T lymphocytes (69–71). On the other hand, CD73 expression marks selected T cell lineages, being found mainly on naïve T cells and in a smaller proportion of memory CD8^+^ cells. While decreasing upon T cell activation, CD73 is high in conditions of chronic inflammation where it associates with a memory T cell phenotype (72).

The finding that CD39 and CD73 are co-expressed on Treg cells (73), highlighted the central role of these ectonucleotidases in driving immune suppression through ADO generation, as witnessed by the observation that CD39^−/−^ mice show significantly impaired suppressive properties in a model of allograft rejection *in vivo* (73). Several studies also suggested that CD39 expression on Tregs suppresses IL-17 production, either preventing trans-differentiation of Tregs into Th17 or endowing already differentiated Th17 cells with an immunosuppressive phenotype (74, 75). At variance with CD39, CD73 is present only in a small proportion of circulating Tregs, but it can be induced upon activation and in conditions of hypoxia. In a study from Stagg et al., it was shown that CD73^−/−^ Tregs are less tumor-supportive than the WT counterpart (76), indicating a role for CD73 and suggesting that its expression is possibly modulated and induced upon Tregs activation. It was also proposed that Tregs are provided with ADO by paracrine interactions with neighboring CD73-expressing cells or through exosome-mediated exchanges (77). ADO, in turn, modulates Treg functions by promoting their proliferation and by increasing CTLA-4 and PD-1 expression, thus ultimately enhancing immunosuppressive potential (78) (Table 1). Increasing evidence suggests that exosome-mediated enzyme exchange could be a more general mechanism, exploited also by tumor cells to provide bystander cells with enzymes creating local conditions of immunosuppression and a tumor-friendly environment (79, 80).

CD39 and CD73 exert their pro-tolerogenic effects on the myeloid compartment as well. For instance, whereas extracellular ATP favors dendritic cells (DC) maturation and activity, by upregulating co-stimulatory molecules as CD38, CD83, CD86, and IL-12 secretion (81), ADO promotes tolerance by increasing IL-6 and IL-10 secretion and expression of immunosuppressive proteins such as indoleamine 2,3-dioxygenase (IDO), TGFβ, and arginase-2 (82). Consistently, anti-inflammatory macrophages with an M2 phenotype co-express CD39 and CD73 and show a higher ATP/AMP hydrolysis compared to pro-inflammatory M1 macrophages, in contrast characterized by low levels of both enzymes (83). This finding suggests that M2 population is accompanied by increase ATP conversion to ADO, which further modulates macrophage functions toward tolerance as witnessed by ADO-mediated increase of M2 markers and enhancement of IL-10 driven responses (84).

Taken together, these observations highlight the fine tuning underneath the regulation of immune system responses. Similar to other biological mechanisms that are derailed in tumors, expression of CD39 and CD73 is often deregulated in neoplastic cells, which take advantage of an immunosuppressive environment that facilitates tumor growth.

## Expression and Functional Role of Ectonucleotidases in Hematological Cancers

The main aim of this review is to discuss the role of ectonucleotidases in hematological malignancies. Wandering around literature, it comes to light that these molecules may have a role as simple markers to distinguish specific cellular populations or subset of patients characterized by a different prognosis, while in other cases they are deeply involved in the pathogenesis of the disease and in molding the tumor microenvironment, critically contributing to leukemic cells proliferation and survival. Similar to solid tumors, leukemia, lymphoma, and myeloma cells establish critical interactions with surrounding non-tumor cells. These interactions take place both in the blood circulation and in protected niches and provide extra stimuli that influence cell behavior. Those occurring in the environmental niches are mostly critical to drive disease progression and chemoresistance, and leukemic cells themselves progressively re-shape the environment to further support their growth while eluding immune surveillance.

Independently on their role, ectonucleotidases have been explored as therapeutic targets, representing in some instances, a winning strategy. In the following sections, we will go through the mechanisms through which NAD- and ATP-dismantling enzymes shape leukemic cells intrinsic features as well as tumor microenvironment in hematological malignancies, exploring also their applications useful for the clinical practice. Targeting strategies will be discussed in a dedicated paragraph.

### NAD^+^ Ectonucleotidases as Prognostic Markers

**Figure 2:**
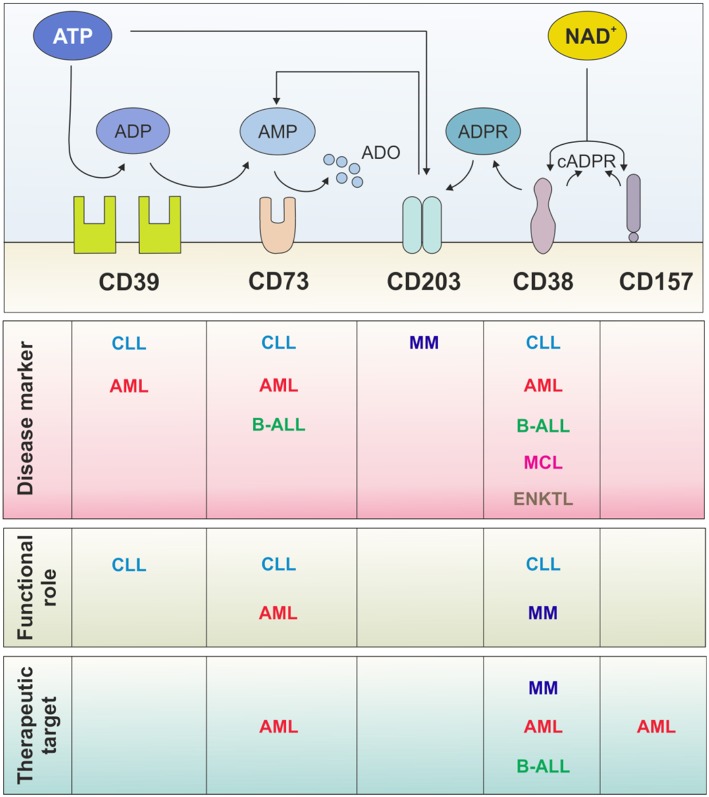
Multiple roles of ectonucleotidases in hematological malignancies. Surface NAD^+^- and ATP-dismantling enzymes have been widely exploited as disease markers for patient stratification and with prognostic relevance. In contrast, few studies, mainly in CLL and AML, address their functional role in the disease biology. Independently of their role, they may represent therapeutic targets: CD38 is a leading example with monoclonal antibodies already in the clinical practice. Several strategies have been designed to target ATP-dismantling enzymes, mainly in solid tumors, and conceivably should be exploited in hematological malignancies in the near future. CLL, chronic lymphocytic leukemia; MCL, mantle-cell lymphoma; B-ALL, B-acute lymphoblastic leukemia; AML, acute myeloid leukemia; ENKTL; extranodal natural killer/T cell lymphoma; MM, multiple myeloma.

CD38 is expressed in several hematological malignancies, including acute B lymphoblastic leukemia (B-ALL) (85), acute myeloid leukemia (AML) (85–87), mantle cell lymphoma (MCL) (88), chronic lymphocytic leukemia (CLL) (89, 90), multiple myeloma (MM) (91) and NK/T cell leukemia (T-ALL) (92, 93) (Figure 2).

The role of CD38 has been widely explored and defined in CLL, and to a lesser extent in MM, whereas little is known about its molecular and biological significance in other malignancies, where it is mainly reported as a negative or positive prognostic indicator.

#### CD38, CD157, and CD203a in AML and ALL

Several years ago, Keyhani and colleagues reported CD38 expression as a positive predictor of disease outcome in previously untreated adult AML and ALL, where CD38 levels correlate with prolonged complete remission and overall survival and represent and independent positive prognostic marker (85). In line with these data, more recently, several papers reported that relapse of Philadephia chromosome positive ALL (Ph^+^ ALL) patients is due to the persistence of leukemia-initiating cells (LICs), a self-renewing CD34^+^CD38^−^CD58^−^ population, capable of initiating human leukemia in immune-deficient mice (94–97). Higher LICs frequencies at diagnosis are predictive of unfavorable prognosis and clinical outcome in AML (98–100) and are considered an independent risk factor for relapse (101). This holds true also in childhood ALL, where a high proportion of CD34^+^CD38^−^ cells negatively correlates with disease outcome and represents a useful marker for patients' risk stratification (102).

At variance with CD38, CD157 presents a more restricted pattern of expression in hematological malignancies, being present only in B-ALL and AML, where it serves as target for selective monoclonal antibodies (103). Specifically, in ALL CD157 was reported as an informative surface marker to discriminate leukemic from normal cells, suggesting its potential application to monitor residual leukemia cell during treatment regimens (104).

In contrast, despite being occasionally reported in solid tumors (105), little is known about pattern of expression of CD203a in hematological malignancies.

#### CD38 in CLL and MM

Twenty years ago, in the attempt to find reliable markers to distinguish among different subset of patients, the association between CD38 expression by CLL cells and a more aggressive behavior was reported for the first time (106). Several independent studies have subsequently confirmed the role of CD38 as a negative prognosticator for this disease and correlated its elevated expression with several adverse prognostic factors such as advanced disease stage, higher incidence of lymphadenopathy, high-risk cytogenetics, shorter lymphocytes doubling time (LDT), shorter time to initiation of first treatment (TFT) and poorer response to therapy (107–111).

Combining CD38 with other prognostic markers, such as Zap70 and CD49d, provides complementary prognostic information. Indeed, flow cytometry analyses on large cohorts of CLL patients indicated that co-expression of CD38 (30% cut-off) with Zap70 (20% cut-off) or with CD49d (30% cut-off) is associated with an unfavorable clinical course (112, 113). A close association between CD38 expression and elevated percentages of Zap70 and Ki-67 has also been highlighted, confirming the attitude of CD38^+^ clones to enter cell cycle (89). The finding of deleterious chromosomal abnormalities such as 11q and 17p deletions or trisomy 12 in patients with a CD38^+^ clone is also consistent with an enhanced proliferative potential (114–116) and a higher degree of clonal evolution (117, 118).

CD38 is highly expressed by MM cells although the characterization of its pathophysiological role in the disease is still an open field of research. Immunophenotyping data indicate that the combination of CD38 and CD138, another known marker of fully differentiated plasma cells, is a validated strategy to identify CD45^+^ myeloma and separate CD20^+^ myeloma from B-cell lymphoma. Moreover, plasma cells immunophenotyping might be useful for detecting minimal residual disease. Given its constituitve expression on plasma cells, it is not surprising that CD38 became an attractive target for CD38^+^ diseases.

#### CD38 in Lymphomas

In MCL, expression of CD38, together with CD20 and CD23, represents a robust marker to identify leukemic cells (88, 119). In addition, CD38 represents a marker to distinguish nodal (~94% of CD38^+^ cases) and non-nodal (~48% of CD38^+^ cases) MCL, with expression correlating with a shorter median survival in several independent series of MCL cases (120, 121).

A similar role for CD38 as prognostic marker was reported for extranodal natural killer/T cell lymphoma (ENKTL), an Epstein-Barr virus associated lymphoma relatively rare in western countries, but more diffuse in the Asian area. The majority of ENKTL cases are CD38^+^ and a stronger expression of this molecule significantly correlates with an inferior outcome, while a weak expression is associated with an increased complete response rate, opening to the possibility of using CD38 as a therapeutic target (93, 122).

### Functions of NAD^+^ Ectonucleotidases in Hematological Malignancies

#### CD38 in AML

A still open and controversial point concerns the role of CD38 in acute promyelocytic leukemia (APL), the M3 subtype of AML, which is characterized by the presence of the chimeric gene promyelocytic leukemia-retinoic acid receptor α (*PML-RARA*) (123) and by an arrest of leukocyte differentiation at the promyelocyte stage. Several clinical regimens combining anthracyclines, arsenic trioxide and retinoic acid (RA) induce differentiation and apoptosis of leukemic cells, resulting in high rates of durable remission in most patients (123, 124). CD38 is one of the earliest markers of RA response: its levels increase exponentially, starting 2–3 h after RA administration (125, 126). Hence, granulocytes differentiated from APL blasts express very high levels of CD38, at variance with their normal counterpart. A recent paper investigated the potential role and impact of CD38 in APL concluding that this molecule is not necessary for RA-induced differentiation. By using selective blocking substrates of the enzymatic activity and CRISPR/Cas9-mediated CD38 truncation, the authors showed that the molecule and its enzymatic activities are dispensable to the differentiation process (127). In conclusion, while the physiologic significance of this aberrant expression remains unclear, it was suggested that CD38 can play a role in a complication of RA therapy known as retinoic acid syndrome (RAS). This complication, which arises soon after RA administration, is characterized by fever, dyspnea and pulmonary edema. One hypothesis to explain this phenomenon involves adhesion of newly differentiated CD38^+^ granulocytes to lung endothelial cells expressing CD31, the CD38 ligand, and resulting in the local production of inflammatory cytokines, apoptosis of endothelial cells and eventually contributing to the development of RAS (128) (Figure 2).

#### CD38 in CLL

Possibly due to intrinsic advantages, such as the high frequency of the disease and the presence of leukemic cells in the peripheral blood, CLL is the leukemia where the functions of CD38 have been studied more in depth.

The first evidence linking CD38 expression to functional responses in leukemic cells came from the observation that CD38^+^ clones showed a more robust response to IgM stimulation compared to negative counterparts (129–131). CD38 ligation with antibodies induced a signaling cascade, including phosphorylation of Zap70 and the MAP kinases, and leading to a more pronounced proliferation and the acquisition of immunoblast like features (132, 133).

Analysis of gene expression profiling after CD38 engagement highlighted migration as one of the most activated biological process (134). Consistently, when exposed to CXCL12, the main chemokine involved in CLL re-circulation from blood to lymph nodes through the binding to CXCR4 (135), CD38^+^ clones exhibited a more pronounced ability to migrate and to activate a selective signaling pathway that the counterpart (136). Lentiviral-induced expression of CD38 in negative primary CLL cells restored their ability to actively respond to the chemokine stimulus (136, 137). In addition to migration, CD38^+^ CLL cells showed more efficient integrin mediated adhesion and matrix digestion. The molecular mechanism behind this functional cooperation resides in their closed physical proximity on the CLL membrane, indicating the existence of a supra-molecular hub that tunes chemotactic responses, adhesion and ability to invade tissues (138, 139).

Within this hub, the likely functions of CD38 is to provide Ca^2+^-mobilizing compounds, mainly ADPR, through NAD^+^ hydrolysis. The implication is that inhibition of the enzymatic activity, impairs CLL chemotaxis, adhesion and *in vivo* homing, by trapping leukemic cells in the blood, preventing their localization in growth-permissive niches and increasing their sensitivity to chemotherapy (140).

#### CD38 in MM

MM is a tumor highly dependent on the BM microenvironment, which functions in a supportive way to promote proliferation, survival, and migration (141). One mechanism through which MM cells communicate with the microenvironment is via microvesiscles trafficking. Immunophenotypic characterization of these particles showed that they express high levels of CD38, together with other ectoenzymes, including CD203a, CD73, and CD39, which show functional activities thereby increasing ADO levels in the niche, ultimately contributing to microenvironment remodeling (142).

Recent data indicate that besides microvesicles, stromal cells from the environment can also deliver mitochondria to MM, contributing to the metabolic adaptation necessary to sustain the high proliferation rate of cancer cells and their homing capability. This organelle transfer has been reported also in MM, where it relies on CD38 and on non-malignant BM stromal cells acting as donors. The use of blocking anti-CD38 antibodies significantly impairs mitochondrial transfer from stromal to MM cells, in turn impacting on their viability (143).

In addition, CD38 is highly expressed also on the Treg fraction of MM patients compared to healthy subjects, and its levels correlate with the suppressive function of Tregs contributing to disease progression and expansion (144).

### ATP Ectonucleotidases as Disease Markers

Similar to solid tumors, deregulation of CD39 and CD73 was also described in hematological malignancies, both of lymphoid and myeloid series. Expression of CD39 and CD73 is generally associated to the development of an immune tolerant environment, contributing to disease expansion. For this reason, levels of these ectonucleotidases, both on leukemic and bystander cells, are often exploited as disease markers or prognosticators. In some instances, the role of CD39 and CD73 has been functionally characterized unraveling a direct contribution in disease pathogenesis and outcome.

#### CD39 and CD73 in ALL and AML

Monitoring the expression of CD39 and/or CD73 is a useful marker also in B-ALL and AML, although the role of nucleotide dismantling in these diseases is far less characterized than in CLL.

In a genome-wide analysis that compared 270 newly diagnosed ALL patients to normal B cell progenitors, CD73 was identified as a differentially expressed molecule, both at the transcript and at the protein levels (145). Flow cytometry validation identified CD73 as a useful marker to monitor minimal residual disease (MRD) as its expression correlated quite precisely to that of molecular markers already in use to predict ALL relapse. Specifically, CD73 is upregulated in B cell precursors of ALL patients and its expression is not down-modulated upon treatment thus making it a reliable marker to identify residual ALL cells resistant to therapies and potentially responsible for future relapses (146, 147).

AML is mainly characterized by aberrant differentiation of myeloid progenitors and impaired control of tumor growth likely due to the immunosuppressive environment created by leukemic blasts, leading to Tregs expansion (148). A study from Dulphy et al. reported that both Tregs and AML blasts express CD39 at higher levels compared to healthy subjects. The authors suggest that Tregs expansion and delocalization, together with ectonucleotidase expression and activity both on Tregs and on AML blasts, could contribute to immune escape of AML ultimately favoring leukemia growth (149). In support of a role for ATP dismantling in eliciting immune suppression in AML, another paper from Lecciso and colleagues reported that AML cells release ATP in response to chemotherapeutics and that through this they modulate dendritic cells and Tregs functions toward tolerance by upregulating CD39 expression on these subsets (150). In contrast, a very recent paper reported that CD73 expression on CD8 T cells favors a reawakening of immune response at variance with CD73^−^/CD8^+^ cells that exhibit a more exhausted profile (151). Beside its role in modulating immune system, CD73 may directly impact on leukemic cell proliferation under the control of CEBPA. In CEBPA mutant AML, CD73 is among the most upregulated genes suggesting that the adenosinergic axis may contribute to AML aggressiveness. Accordingly, silencing of CD73 significantly delays leukemia development in mice thus establishing a tumor-promoting role of this enzyme in leukemia progression *in vivo* (152).

It is therefore becoming clear that upregulation of CD39 and CD73 marks leukemic cells characterized by a more aggressive behavior likely resulting from a more permissive environment or a more efficient adaptation. The pathogenic role of nucleotide dismantling and its contribution to disease evolution has been characterized in relatively few disease models, on top of which there is CLL (Figure 2).

#### CD39 and CD73 in CLL

In CLL, several studies observed that CD39 expression is markedly increased in circulating leukemic cells compared to matched healthy subjects, and levels are further increased when looking at cells residing in the LN, representing a CLL sanctuary. Specifically, the proliferating Ki-67^+^ CLL fraction expresses CD39 at higher levels compared to the resting population that showed consistently weaker expression, suggesting a role in disease aggressiveness (153, 154). Besides leukemic cells, CLL LNs are rich of stromal components that are intensely CD39^+^, including T lymphocytes. The comparative analysis of T cell subsets highlighted that, overall, CLL patients have a higher percentage of CD39^+^ T lymphocytes than controls, both in the CD4^+^ and in the CD8^+^ compartment (155). Similar to CD39, CD73 expression is also associated with the proliferating CLL fraction that is in close contact with CD4^+^CD25^+^ Tregs although with more variability (156). CD73 expression characterize roughly one third of CLL patients, showing also intra-leukemic heterogeneity. This means that, despite virtually all CLL cells could degrade ADP to AMP, as they constitutively express CD39, not all of them can produce ADO at appreciable levels. It was observed that a cut-off of ≥30% CD73^+^ cells, is needed and ectoenzyme expression is in turn tightly regulated by local conditions, most important hypoxia (155, 157). Both CD39 and CD73 were investigated for CLL patients' prognostic classification. CD39 expression on the T cells fraction strongly associates with a more advanced disease stage and is considered predictive of treatment requirement, as significantly shorter TTFT was observed in patients with CD39^high^ T cells compared to CD39^low^ (158).

CD73 on leukemic B cells associates with other negative prognostic markers, such as CD38 and Zap70. It was proposed that CD73 expression identifies a leukemic subset of cells characterized by (i) an increased recirculation to and from the lymphoid niche, (ii) a more aggressive clinical behavior, (iii) a higher cellular turnover and (iv) associates with time to disease progression after fludarabine treatment (155, 159).

### ATP Ectonucleotidases in the Pathogenesis and Outcome of Hematological Malignancies

In the tumor microenvironment, ATP is abundantly released in the extracellular space, likely as the result of metabolic stress due to hypoxia and pro-inflammatory signals. Even if it could potentially recruit immune cells to fight against the tumor, ectonucleotidases ensure a rapid conversion of ATP to ADO, which accumulates in the extracellular space, contributing to skewing immune cells toward tolerance, supporting tumor growth instead (160). The role of ATP ectonucleotidases is mostly connected to the control of immunomodulatory activities and has been inferred by using drugs that modulate ADO production and signaling. Overexpression of both ATP-dismantling ectoenzymes and ADO receptors might carry negative prognostic relevance in hematological malignancies, marking aggressive disease and immunosuppression (161).

In a model of FL, hypo-responsiveness of infiltrating T cells could be partially reversed by blockade of adenosine receptors or inhibitors of CD39 activity. Accordingly, CD39 was upregulated in T cells residing in FL lymph nodes suggesting that ATP-dismantling and ADO signaling could be responsible for T cell anergy favoring FL expansion (162).

In CLL there is an intense cross-talk between leukemic and non-tumor bystander cells in microenvironment-associated structures (e.g., LN and BM), where adenosinergic signaling is integrated in the complex network of cell-cell contacts and soluble mediators driving leukemia (14, 163). CLL cells are equipped with both CD39/CD73 and A2A ADO receptors, indicating that adenosinergic signaling operates through both autocrine and paracrine effects. On the CLL side, high ADO levels contribute to keep leukemic cells in the growth-supportive environment of the LN, where they are recruited by chemokine signals (155). Furthermore, ADO up-regulates anti-apoptotic mediators, limiting the efficacy of chemotherapy. Consequently, in the LN environment, leukemic cells are protected from spontaneous and drug-induced apoptosis, maintaining a leukemia reservoir that fuels disease progression (164).

On the other hand, ADO acts on immune system cells, polarizing them toward tolerance, further sustaining leukemic cell expansion. The molecular mechanisms behind this effect involve the activation of the HIF1α genetic program (165), which directly upregulates ectonucleotidases expression and ADO signaling (166), as observed in other disease conditions (167, 168). Experimental evidence confirms that CD73 expression is significantly increased under *in vitro* hypoxic conditions, both at the mRNA level (*NT5E*) and on the surface of nurse-like cells (157), a type 2 (M2) macrophage population. When derived *in vitro* under low oxygen tension, NLCs significantly upregulated ADO production, fueling A2A signaling of surrounding cells and activating autocrine signaling through MAPK, PI3K, and NF-κB (157). ADO receptor signaling further contributes to M2 macrophage polarization by upregulating the transcription factor IRF4 and the tryptophan-metabolizing enzyme IDO, both M2 macrophages markers (169), and increasing synthesis of IL-6 (157).

Moreover, hypoxia-mediated upregulation of CD73 and of ADO generation also influences metabolic adaptation of the T lymphocytes surrounding the tumor, inducing a switch toward glycolysis, with robust upregulation of glucose and lactate transporters and of lactate dehydrogenase (LDHA) and pyruvate kinase 2 (PKM2). This metabolic conversion skews T cells functions to a regulatory phenotype, as indicated by increased expression of *FOXP3, PD-1, IL-10*, and *VEGFA* and the down-modulation of interferon γ (*IFNG*) mRNA. These effects can be recapitulated upon pharmacological modulation of A2A activation (157). Similar outcomes are observed on the leukemic fraction, in line with evidence that metabolic adaptation of tumor cells upon oxygen deprivation rapidly fosters energy production via glycolysis through HIF1α-mediated transcriptional control (170). In keeping with the immunomodulatory effects registered in the T cell compartment, metabolic adaptation induced by the hypoxia-ADO axis on leukemic B cells tunes CLL cytokine production and release and favors the acquisition of a B-regulatory phenotype, as defined by a marked upregulation of IL-10 (171). These findings link hypoxia and CD73-generated ADO in a common axis aimed at re-shaping a tumor-favorable niche with tumor-supportive and immunosuppressive features (157) (Figure 2).

## Ectonucleotidases as Therapeutic Targets

Interrupting deregulated circuits promoting leukemia expansion, while awakening the immune surveillance against tumor cells, represents the “fil rouge” of target therapy approaches. In this scenario, nucleotide-dismantling enzymes represent good candidates as they play multifaceted roles, thus offering multiple levels of targeting. First, given their specific pattern of expression, they may serve as markers to selectively tag leukemic cells and deliver therapeutic agents while limiting off-target effects. Second, since they operate in a coordinated cascade of events, inhibition of one of them is sufficient to block the downstream processes. Lastly, they play several roles in tumor biology, so interfering with them opens the possibility to modulate immunosuppression. However, at least in hematological malignancies, clinical trials have just reached the first level, i.e., using antibodies to ectonucleotidases to specifically target a disease population.

Daratumumab, a humanized anti-CD38 IgG1 mAb, is the forefather and the most advanced immunotherapeutic agent, which was approved for clinical use in 2015 (172). Its primary application is in multiple myeloma (MM) where CD38 is highly and uniformly expressed, but it is also being evaluated in lymphomas, including diffuse large B cell lymphoma, FL and mantle cell lymphoma, in AML and in ALL (87, 173). Generally well-tolerated, daratumumab induces partial or complete responses in ~30% of heavily pretreated MM patients, when used as monotherapy and in this setting received its first approval in 2015. However, based on its distinct mechanisms of action, favorable toxicity profile and single-agent activity, daratumumab is an attractive partner in combination regimens. Significant responses and prolonged progression-free survival can be achieved in combination with immunomodulatory agents or proteasome inhibitors (174, 175). For these reasons, daratumumab is now approved by the FDA and EMA in combination with lenalidomide or bortezomib in MM patients who have received at least one prior therapy. Several clinical trials are ongoing both in the United States and Europe, evaluating the efficacy of this mAb with other drug combinations or as frontline therapy (175). The documented mechanisms of action include antibody-dependent cell cytotoxicity (ADCC), complement-dependent cytotoxicity (CDC), antibody-dependent cellular phagocytosis (ADCP), inhibition of CD38 enzymatic activities and induction of apoptosis in a caspase-dependent manner (176–178).

More recently new anti-CD38 antibodies are being developed, including isatuximab, a chimeric mouse/human Ab (179), and MOR202, a fully human Ab (180), underlying the interest of the medical community.

Besides mAbs, an alternative approach to target CD38 is the use of enzyme inhibitors. For example, flavonoids, such as kuromanin, bind to critical residues in the catalytic site competing with the substrates (140, 181). However, these strategies are still very far from clinical applications.

Targeting leukemic cells exploiting ectonucleotidases as surface markers is an approach pursued also with CD157. *In vitro* and *ex vivo* preclinical studies in AML and ALL show that anti-CD157 antibody, namely OBT357/MEN1112, has highly specific cytotoxicity against leukemic cells (103, 182). The antibody is currently being tested in a clinical trial for patients with relapsed or refractory AML.

In contrast, targeting of CD39 and CD73 in hematological malignancies remains an unexplored field despite promising results obtained in solid tumors, where modulation of their enzymatic activities is achieved by multiple strategies. Several CD39 inhibitors, including ARL67156 and POM-1, have shown efficacy in animal models of follicular lymphoma, sarcoma, or murine melanoma, resulting in a partial overcome of T-cell hypo-responsiveness to stimulation (162), increased therapeutic response to chemotherapeutic agents (183), or inhibition of tumor growth (184), respectively. Anti-CD39 antibodies, which also block the enzymatic activity, seem to be effective in different tumors, such as sarcoma and ovarian cancer (185–187). The efficacy of targeting ATP-dismantling enzymes as an anti-tumor therapy derives also from studies in breast cancer models where treatment of mice with an anti-CD73 antibody partially prevented lung metastases (188). Accordingly, a therapeutic anti-CD73 antibody, MEDI9447 is currently in clinical trials in solid cancer patients (NCT02503774; NCT03611556) (189). Potentially promising in a translational perspective is the compound a,b-methylene-ADP (APCP), the most potent competitive CD73 inhibitor, although no clinical trials are currently ongoing (190, 191).

More recently, nucleotide metabolizing enzymes have been explored as targets for the generation of nanobodies, soluble antibodies that correspond to the variable domain fragments derived from the heavy chain. A pool of nanobodies targeting CD38 is currently undergoing selection and functional characterization. Stemming from these results, nanobodies against CD39, CD73, and CD203 are likely to be designed (192) (Figure 2).

## Conclusions

In this review, we have discussed basic knowledge on ectonucleotidases, addressing their role in hematological malignancies where they can be surface markers and, in some instances, they can directly contribute to leukemia expansion. The contributing role of ATP and NAD-dismantling enzymes is underlined by their involvement in the regulation of homing processes and in favoring local conditions of immunosuppression.

The emerging picture is that they represent good targets for novel therapeutic strategies. However, several unaddressed questions remain, including the convergence and cross-talk of these pathways toward the production of adenosine, the functional characterization and impact in leukemia other than CLL and the feasibility and relevance of their inhibition. This holds true mainly for the adenosinergic axis for which most of the current knowledge on therapeutic strategies comes from solid tumors. Efforts are being made to design inhibitors of adenosine signaling, which include compounds targeting A2A (CPI-444, PBF-509 and AZD4635) currently in Phase I/II clinical trials (193–195).

At the light of the major effects obtained with Daratumumab in MM, it is conceivable that in the near future additional “bullets” targeting ectonucleotidases will reach the market as adjunctive tools to conventional chemotherapy.

## Author Contributions

All authors listed have made a substantial, direct and intellectual contribution to the work, and approved it for publication.

### Conflict of Interest

The authors declare that the research was conducted in the absence of any commercial or financial relationships that could be construed as a potential conflict of interest.
